# Relationship between glycemic variability and renal function progression in patients with diabetic nephropathy: A retrospective cohort study

**DOI:** 10.1097/MD.0000000000047119

**Published:** 2026-01-30

**Authors:** Ling Yang, Bo Zhou, Rensheng Deng

**Affiliations:** a Endocrinology Department, The Lichuan Ethnic Hospital of Traditional Chinese Medicine, Lichuan City, Hubei Province, China; b Endocrinology Department, Qianjiang Central Hospital, Qianjiang City, Hubei Province, China; c Endocrinology Department, Enshi Tujia and Miao Autonomous Prefecture Central Hospital, Enshi Tujia and Miao Autonomous Prefecture, Enshi, Hubei Province, China.

**Keywords:** continuous glucose monitoring, diabetic kidney disease, estimated glomerular filtration rate, glucose variability, renal function decline, risk assessment

## Abstract

Diabetic kidney disease (DKD) is the leading cause of end-stage renal disease in diabetes. Beyond chronic hyperglycemia, glycemic variability (GV) has emerged as a contributor to diabetic complications, but its prognostic role in DKD is unclear. This single-center retrospective cohort study enrolled 156 patients with DKD and followed them for a median of 36 months. GV was assessed using the coefficient of variation and patients were stratified into quartiles. The primary endpoint was a composite of ≥40% decline in estimated glomerular filtration rate (eGFR), end-stage renal disease, or kidney-related death. Cox regression and linear mixed-effects models were applied. Patients in the highest GV quartile had a significantly higher incidence of renal events than those in the lowest (43.6% vs 12.8%, *P* for trend = .015), corresponding to nearly a 3-fold increased risk (hazard ratio = 2.79, 95% confidence interval: 1.18–6.62). Each 1-standard deviation increase in coefficient of variation was associated with a 22% to 30% higher risk of adverse outcomes (all *P* < .05), and mixed-effects models confirmed a dose–response relationship with accelerated eGFR decline. Subgroup analyses showed stronger associations in patients with glycated hemoglobin A1c ≥7.0%, baseline eGFR <60 mL/min/1.73 m², and those not treated with SGLT2 inhibitors. In conclusion, higher GV is independently associated with adverse renal outcomes and faster eGFR decline in DKD. Incorporating GV into risk stratification may support individualized glycemic management and help delay disease progression.

## 1. Introduction

Diabetic kidney disease (DKD) is among the most prevalent and serious microvascular complications of type 2 diabetes. Clinically, it is characterized by persistent proteinuria, elevated blood pressure, and a progressive decline in estimated glomerular filtration rate (eGFR), ultimately advancing to end-stage kidney disease (ESKD) (a leading indication for dialysis and kidney transplantation).^[1]^ With the global rise in diabetes prevalence, DKD has emerged as a major public health challenge, placing considerable strain on healthcare systems due to the growing demand for renal replacement therapy. Consequently, delaying disease progression, improving patient outcomes, and alleviating healthcare costs have become central goals in both clinical management and research. Conventional glycemic indices, such as glycated hemoglobin (HbA1c) and fasting plasma glucose, primarily reflect average glucose levels but fail to capture short-term fluctuations in daily life. Moreover, they do not adequately represent the risk of target organ damage driven by recurrent glycemic excursions.^[2]^

In recent years, glycemic variability (GV) has been increasingly recognized as an important complementary marker in glucose management. GV reflects both the extent and frequency of blood glucose fluctuations within a given period and is commonly quantified using standard deviation (SD), coefficient of variation (CV), mean amplitude of glycemic excursions (MAGE), and time in range (TIR). Unlike average glucose indices, GV provides a more sensitive measure of repeated exposure to hyperglycemic peaks and hypoglycemic episodes. Growing evidence suggests that GV contributes independently to the development and progression of diabetic complications. Epidemiological studies have linked elevated GV to diabetic retinopathy, cardiovascular events, and all-cause mortality,^[3–5]^ underscoring its substantial clinical relevance.

With respect to kidney disease, an increasing body of research has examined the link between GV and chronic kidney disease (CKD) progression. In the broader diabetic population, long-term HbA1c variability has been identified as an independent predictor of renal outcomes. For example, a study of 3466 patients showed that different measures of HbA1c variability consistently predicted renal function decline, with individuals in the highest quartile having nearly a 7-fold greater risk.^[6]^ Similarly, a cohort analysis using the UK Clinical Practice Research Datalink reported a strong association between high HbA1c variability and the incidence of ESKD.^[7]^ More recent studies in patients with type 2 diabetes and CKD have confirmed that HbA1c variability is positively correlated with key adverse endpoints, including sustained eGFR decline and worsening proteinuria.^[4]^ Zhang et al further demonstrated that patients with greater HbA1c fluctuations during follow-up were more likely to develop progressive proteinuria and accelerated eGFR loss.^[8]^ Moreover, a meta-analysis pooling multiple observational studies reinforced the robust positive relationship between GV and CKD progression.^[9]^

Despite these findings, current evidence remains limited. Most studies have been conducted in the general diabetic population, with relatively little data available for patients with established DKD. In addition, the majority of prior work has assessed GV using HbA1c variability, which primarily reflects long-term glycemic averages and fails to capture short-term fluctuations. Metrics derived from continuous glucose monitoring (CGM), such as TIR and CV, provide a more comprehensive assessment of glucose dynamics, yet their prognostic value in DKD has not been adequately investigated.^[10]^ Moreover, existing studies have rarely incorporated sensitivity analyses, subgroup comparisons, or dose–response assessments, limiting the ability to clarify GV’s true impact on renal function across diverse clinical contexts. Critically, the association between GV and continuous measures of renal deterioration (such as the annual decline in eGFR and the yearly change in urine albumin-to-creatinine ratio [UACR]) remains insufficiently explored, hindering a deeper understanding of its underlying pathophysiological mechanisms.

Against this background, this study systematically investigated the association between GV and renal function progression in patients with confirmed DKD. In addition to assessing composite renal outcomes (defined as a ≥40% decline in eGFR, development of end-stage renal disease, or kidney-related death) we evaluated longitudinal changes in eGFR decline rates and UACR trajectories to capture the dynamic evolution of renal function. Using multivariable Cox regression, linear mixed-effects modeling, sensitivity testing, and subgroup analyses, we aimed to confirm the robustness of our findings and explore potential dose–response relationships between GV and adverse outcomes. We hypothesized that higher GV would be associated with accelerated renal deterioration and a greater risk of adverse renal events. These findings may help address current evidence gaps, providing novel insights and a rationale for early identification of high-risk patients, more individualized glycemic management, and strategies to delay DKD progression.

## 2. Methods

### 2.1. Study design and subjects

This study was approved by the Ethics Committee of Enshi Tujia and Miao Autonomous Prefecture Central Hospital.This single-center retrospective cohort study was conducted at our institution between January 2020 and December 2024. Eligible participants met the following criteria: a confirmed diagnosis of type 2 diabetes mellitus with evidence of chronic kidney disease, defined as eGFR <90 mL/min/1.73 m² or abnormal proteinuria; and availability of at least 6 consecutive months of blood glucose monitoring data sufficient for calculating GV indices. Patients were excluded if they had renal impairment attributable to other primary glomerular diseases or systemic conditions; a history of kidney transplantation or dialysis; or incomplete clinical data or a follow-up period of >6 months. Because this was a retrospective cohort study, the sample size was not estimated in advance; instead, all eligible patients within the study period were included.

All participants were followed from baseline until the occurrence of a composite renal endpoint, death, or December 31, 2024, whichever came first. Owing to the retrospective design, informed consent was not required.

### 2.2. Assessment of glucose variability

GV indices were derived from CGM during hospitalization and outpatient glucose monitoring records. To ensure data reliability, only complete monitoring periods of ≥14 consecutive days were included. In line with established methodologies, the following metrics were calculated:

- SD (mmol/L): dispersion of glucose values;
- CV (%): SD divided by mean glucose × 100%;
- MAGE (mmol/L): average magnitude of glucose fluctuations;
- TIR (%): proportion of time spent within 3.9 to 10.0 mmol/L.

Patients were stratified into quartiles (Q1–Q4) according to CV. CV was selected as the primary GV parameter for analysis, while the other indices were applied in sensitivity analyses.

### 2.3. Covariate collection

Baseline information was obtained from electronic medical records, including demographics (age, sex, body mass index), diabetes duration, blood pressure, HbA1c, and fasting plasma glucose. Medication data covered insulin, metformin, SGLT2 inhibitors, glucagon-like peptide-1 receptor agonists, and renin–angiotensin–aldosterone system inhibitors. Comorbidities comprised hypertension, dyslipidemia, and cardiovascular disease. Renal assessments included baseline eGFR, UACR, and albuminuria stage (A1–A3).

### 2.4. Outcome measures

The primary outcome was a composite renal endpoint, defined as: a ≥40% decline in eGFR from baseline confirmed by at least 2 consecutive measurements; progression to ESKD, indicated by maintenance dialysis for ≥3 months or kidney transplantation; or kidney-related mortality.

Secondary outcomes included the annual rate of eGFR decline and the yearly change in UACR. Renal function was evaluated at 3- to 6-month intervals during follow-up.

### 2.5. Statistical analysis

Continuous variables with normal distribution were reported as mean ± SD and compared by one-way analysis of variance, while skewed data were summarized as median and tested with the Kruskal–Wallis method. Categorical variables were expressed as n (%) and assessed using the Chi-square or Fisher exact test.

The incidence of composite renal outcomes was assessed with Kaplan–Meier survival analysis and compared across groups using log-rank tests. The relationship between GV and renal function decline was evaluated through Cox proportional hazards models with 3 levels of adjustment: age and sex; additional adjustment for diabetes duration, HbA1c, baseline eGFR, and medication use; and inclusion of all potential confounders.

Sensitivity analyses were performed by substituting alternative GV metrics, including SD, MAGE, and TIR. Subgroup analyses were stratified by HbA1c (<7.0% vs ≥7.0%), baseline eGFR (≥60 vs <60 mL/min/1.73 m²), albuminuria stage (A1 vs A2–A3), and use of SGLT2 inhibitors or insulin. Interaction terms were tested to evaluate potential effect modification.

Missing data were addressed using multiple imputation under the assumption of random missingness. For sensitivity and subgroup analyses, *P*-values from multiple comparisons were adjusted using the false discovery rate method.

A linear mixed-effects model was also applied to evaluate the association between GV and the rate of eGFR decline, accounting for repeated measurements. All statistical analyses were conducted using R software (version 4.2.2; R Foundation for Statistical Computing, Vienna, Austria), primarily with the survival and lme4 packages. A two-sided *P* < .05 was considered statistically significant.

## 3. Results

### 3.1. Baseline characteristics

A total of 156 patients with DKD were included and categorized into quartiles according to GV (Q1–Q4, 39 patients each). Table 1 summarizes the baseline characteristics. The groups were generally similar in age, sex, body mass index, comorbidities, and most medication use. In contrast, higher GV was associated with longer diabetes duration (*P* = .017), higher systolic blood pressure (*P* = .009), and poorer glycemic control, reflected by rising HbA1c and fasting plasma glucose levels (both *P* < .001). GV-related parameters (SD, CV, MAGE) showed progressive increases, while TIR declined consistently across quartiles (all *P* < .001).

**Table 1:** Patient baseline characteristics stratified by GV quartile.

Projects	Total (N = 156)	Q1 (lowest GV) (n = 39)	Q2 (n = 39)	Q3 (n = 39)	Q4 (highest GV) (n = 39)	*P*-value
Age (yr), mean ± SD	62.1 ± 10.8	61.2 ± 10.6	61.7 ± 10.5	62.4 ± 11.0	63.2 ± 11.1	.497
Male, n (%)	97 (62.2)	22 (56.4)	24 (61.5)	25 (64.1)	26 (66.7)	.812
BMI (kg/m²), mean ± SD	27.0 ± 3.9	26.5 ± 3.7	26.8 ± 3.8	27.2 ± 3.9	27.6 ± 4.1	.864
Duration of diabetes (yr), median (IQR)	12 (7–18)	10 (6–16)	11 (7–17)	13 (8–19)	14 (9–20)	.017
Systolic blood pressure (mm Hg), mean ± SD	137 ± 16	134 ± 15	136 ± 15	138 ± 16	141 ± 17	.009
Diastolic blood pressure (mm Hg), mean ± SD	79 ± 10	78 ± 9	79 ± 10	80 ± 10	81 ± 11	.393
HbA1c (%), mean ± SD	7.8 ± 1.2	7.2 ± 1.0	7.6 ± 1.1	8.0 ± 1.2	8.4 ± 1.3	<.001
Fasting plasma glucose (mmol/L), mean ± SD	7.84 ± 1.91	7.20 ± 1.62	7.62 ± 1.78	8.00 ± 1.92	8.56 ± 2.04	<.001
*Glycemic variability indices*						
Glucose SD (mmol/L)	2.11 ± 0.85	1.18 ± 0.28	1.72 ± 0.33	2.28 ± 0.40	3.25 ± 0.70	<.001
Coefficient of variation (CV), %	26.7 ± 8.6	17.2 ± 3.5	23.1 ± 3.6	29.0 ± 4.0	37.4 ± 6.4	<.001
MAGE (mmol/L)	4.8 ± 1.9	2.8 ± 0.8	4.0 ± 0.9	5.4 ± 1.1	6.8 ± 1.6	<.001
Time in range (TIR) (3.9–10.0 mmol/L), %	58 (42–74)	74 (62–83)	64 (50–78)	52 (38–66)	38 (26–52)	<.001
*Medication use*						
Insulin use, n (%)	74 (47.4)	13 (33.3)	16 (41.0)	21 (53.8)	24 (61.5)	.067
Metformin, n (%)	110 (70.5)	30 (76.9)	28 (71.8)	26 (66.7)	26 (66.7)	.766
SGLT2 inhibitor, n (%)	56 (35.9)	17 (43.6)	15 (38.5)	12 (30.8)	12 (30.8)	.607
GLP-1RA, n (%)	25 (16.0)	6 (15.4)	6 (15.4)	6 (15.4)	7 (17.9)	.988
RAAS inhibitor, n (%)	103 (66.0)	24 (61.5)	25 (64.1)	26 (66.7)	28 (71.8)	.876
*Comorbidities*						
Hypertension, n (%)	106 (67.9)	23 (59.0)	25 (64.1)	28 (71.8)	30 (76.9)	.436
Dyslipidemia, n (%)	94 (60.3)	21 (53.8)	23 (59.0)	24 (61.5)	26 (66.7)	.784
Cardiovascular disease, n (%)	36 (23.1)	7 (17.9)	8 (20.5)	9 (23.1)	12 (30.8)	.616
*Baseline renal function*						
eGFR (mL/min/1.73 m²) mean ± SD	68.8 ± 23.5	73.5 ± 22.4	70.6 ± 22.9	66.7 ± 23.7	64.3 ± 24.0	.181
UACR (mg/g), median (IQR)	150 (40–610)	100 (30–410)	125 (36–520)	170 (48–680)	230 (62–810)	.201
Albuminuria category (A1/A2/A3), n (%)	36/58/62	12/15/12	9/17/13	7/14/18	8/12/19	.565

BMI = body mass index, eGFR = estimated glomerular filtration rate, GLP-1RAs = glucagon-like peptide-1 receptor agonists, GV = glycemic variability, IQR = interquartile range, MAGE = mean amplitude of glycemic excursions, RAAS = renin–angiotensin–aldosterone system, UACR = urine albumin-to-creatinine ratio.

### 3.2. Renal outcomes across different GV quartiles

The median follow-up was 36 months. During this period, the incidence of composite renal outcomes increased from 12.8% in Q1 to 43.6% in Q4 (*P* for trend = .015), corresponding to event rates rising from 5.1 to 17.4 per 100 person-years. The median time to event was also significantly shorter in the highest GV group (Q1: 31 months vs Q4: 18 months, *P* = .018). In addition, the annual rate of eGFR decline accelerated with higher GV (Q1: ‐1.8 vs Q4: ‐4.1 mL/min/1.73 m²/year, *P* < .001), while the annual rate of UACR progression also increased steadily across quartiles (*P* < .001) (Table 2).

**Table 2:** Renal outcomes and decline in eGFR by different GV quartiles.

Conclusion	Q1 (lowest GV)	Q2	Q3	Q4 (highest GV)	*P*-value
Composite renal endpoint, n (%)	5 (12.8)	8 (20.5)	11 (28.2)	17 (43.6)	.015
Incidence rate (per 100 person-years)*	5.1	8.2	11.3	17.4	–
Median time to event, months (IQR)	31 (24–40)	28 (20–38)	24 (16–34)	18 (11–28)	.018
eGFR decline rate (mL/min/1.73m²/year, mean ± SD)	‐1.8 ± 1.3	‐2.5 ± 1.6	‐3.2 ± 1.9	‐4.1 ± 2.3	<.001
Annual percent change in UACR, % (median [IQR])	+5 (‐8 to +22)	+11 (‐4 to +30)	+18 (+2 to +43)	+27 (+9 to +61)	<.001

eGFR = estimated glomerular filtration rate, GV = glycemic variability, IQR = interquartile range, UACR = urine albumin-to-creatinine ratio.

### 3.3. Cox proportional hazards regression analysis

Multivariable Cox regression analysis demonstrated a significant association between higher GV and renal function progression (Table 3). Compared with Q1, patients in Q4 had a markedly elevated risk (Model 1: hazard ratio [HR] = 3.56, 95% confidence interval [CI]: 1.53–8.30, *P* = .003; Model 2: HR = 2.79, 95% CI: 1.18–6.62, *P* = .019). In the fully adjusted model, the association persisted as a trend (HR = 2.24, 95% CI: 0.93–5.41, *P* = .072). Moreover, each 1-SD increase in CV was consistently linked to higher risk of adverse outcomes (HR range: 1.22–1.30, all *P* < .05).

**Table 3:** Multivariate Cox regression analysis of glycemic variability and risk of renal function progression.

Exposure factors	Model 1 HR (95% CI)	*P*-value	Model 2 HR (95% CI)	*P*-value	Model 3 HR (95% CI)	Trend *P*-value
Q1 (Reference)	1.00	–	1.00	–	1.00	–
Q2	1.36 (0.54–3.42)	.510	1.32 (0.52–3.31)	.558	1.28 (0.50–3.25)	.610
Q3	2.08 (0.86–5.00)	.105	1.92 (0.79–4.65)	.148	1.78 (0.72–4.42)	.211
Q4 (highest GV)	3.56 (1.53–8.30)	.003	2.79 (1.18–6.62)	.019	2.24 (0.93–5.41)	.072
GV, per 1-SD increase (CV%)	1.30 (1.06–1.61)	.012	1.26 (1.03–1.55)	.026	1.22 (1.01–1.49)	.041

CI = confidence interval, CV = coefficient of variation, GV = glycemic variability.

### 3.4. Sensitivity analysis

Sensitivity analyses yielded results consistent with the primary findings (Table 4). Among the GV metrics, CV showed the most robust association, with each 1-SD increase corresponding to a 22% higher risk of renal function progression (HR = 1.22, 95% CI: 1.01–1.49, *P* = .041). SD, MAGE, and TIR demonstrated similar upward trends, although these did not reach statistical significance.

**Table 4:** Sensitivity analysis of different GV indicators and risk of renal function progression.

GV indicator	Per 1-SD increment, HR (95% CI)	*P*-value	Q4 vs Q1 HR (95% CI)	Trend *P*-value
CV, % (primary)	1.22 (1.01–1.49)	.041	2.24 (0.93–5.41)	.072
Blood glucose SD (mmol/L)	1.18 (0.98–1.43)	.078	2.03 (0.85–4.90)	.111
MAGE (mmol/L)	1.20 (0.99–1.46)	.062	2.12 (0.88–5.10)	.095
Per 10% decrease in TIR	1.13 (0.98–1.32)	.094	1.88 (0.78–4.61)	.156

CI = confidence interval, CV = coefficient of variation, GV = glycemic variability, MAGE = mean amplitude of glycemic excursions.

### 3.5. Subgroup analysis

Subgroup analyses are summarized in Table 5. The association between GV and renal progression was generally consistent across clinical subgroups, with stronger effects observed in patients with HbA1c ≥7.0% (HR = 1.26, *P* = .034), baseline eGFR <60 mL/min/1.73 m² (HR = 1.28, *P* = .043), albuminuria stage A2 to A3 (HR = 1.25, *P* = .022), and those not treated with SGLT2 inhibitors (HR = 1.27, *P* = .022). Nonetheless, no statistically significant interactions were detected (all *P* > .05).

**Table 5:** Relationship between GV (per 1 SD increase) and renal function progression in different subgroups.

Subgroup	Number of events/N	HR (95% CI)	*P*-value	Interaction *P*-value
HbA1c <7.0%	14/72	1.18 (0.90–1.56)	.225	
HbA1c ≥7.0%	27/84	1.26 (1.02–1.63)	.034	.438
Baseline eGFR ≥60	22/88	1.20 (0.95–1.53)	.128	
Baseline eGFR <60	19/68	1.28 (1.01–1.66)	.043	.512
Albuminuria A1	5/36	1.15 (0.77–1.83)	.486	
Albuminuria A2–A3	36/120	1.25 (1.04–1.56)	.022	.361
SGLT2 inhibitor users	10/56	1.16 (0.86–1.66)	.336	
Non-SGLT2 inhibitor users	31/100	1.27 (1.04–1.60)	.022	.477
Insulin users	22/74	1.23 (0.99–1.61)	.063	
Non-insulin users	19/82	1.20 (0.95–1.55)	.136	.811

CI = confidence interval, eGFR = estimated glomerular filtration rate, GV = glycemic variability.

### 3.6. Linear mixed-effects model

The linear mixed-effects model further confirmed the association between CV and eGFR decline (Table 6). Each 1-SD increase in CV was linked to an additional annual reduction of 0.31 mL/min/1.73 m² (*P* = .012). Compared with Q1, eGFR decline rates were accelerated by ‐0.56, ‐1.09, and ‐1.78 mL/min/1.73 m²/year in Q2, Q3, and Q4, respectively (all *P* < .05), indicating a clear dose–response relationship.

**Table 6:** Linear mixed-effects model: relationship between GV and annual eGFR decline rate (sample data).

Exposure factors	β value (eGFR slope, mL/min/1.73 m²/year) (95% CI)	*P*-value
GV, per 1-SD increase (CV%)	‐0.31 (‐0.55 to ‐0.07)	.012
Q2 vs. Q1	‐0.56 (‐1.04 to ‐0.08)	.023
Q3 vs. Q1	‐1.09 (‐1.58 to ‐0.60)	<.001
Q4 vs. Q1	‐1.78 (‐2.35 to ‐1.21)	<.001

CI = confidence interval, CV = coefficient of variation, eGFR = estimated glomerular filtration rate, GV = glycemic variability.

## 4. Discussion

This study systematically examined the association between GV and renal function decline in patients with confirmed DKD. We found that higher GV was significantly associated with increased incidence of composite renal outcomes, faster eGFR decline, and greater annual progression in UACR. Importantly, these associations persisted after adjustment for multiple confounders and demonstrated a clear dose–response relationship. Our findings add novel evidence supporting GV as an independent predictor of renal prognosis in DKD.

Compared with previous research, this study provides several novel contributions. Most earlier investigations examined general diabetic populations, whereas evidence in patients with established DKD has been relatively scarce. Our study not only confirmed the association between HbA1c variability and renal outcomes but also, for the first time, simultaneously evaluated CGM-derived metrics (CV, SD, MAGE, and TIR) in a DKD cohort. The robustness of these findings was supported by consistent results from sensitivity and subgroup analyses. Furthermore, linear mixed-effects modeling demonstrated that GV influences not only renal endpoints but also the rate of eGFR decline, underscoring its sustained impact on the dynamic trajectory of renal function. These observations align with reports from Zhu et al^[4]^ and Zhang et al,^[10]^ as well as the long-term follow-up study by Yan et al,^[11]^ all of which identified HbA1c variability as an important predictor of DKD progression. In addition, our results highlight CV as the most stable and predictive GV metric, consistent with conclusions drawn by Battelino et al^[3]^ and Xu et al.^[12]^

Subgroup analyses indicated that the impact of GV was more pronounced in high-risk populations, including patients with HbA1c ≥7.0%, baseline eGFR <60 mL/min/1.73 m², albuminuria stage A2 to A3, and those not receiving SGLT2 inhibitors. These results suggest that GV may serve as a valuable supplementary marker for risk stratification and individualized management in vulnerable subgroups. Shah et al^[13]^ reported that poor glycemic control is strongly linked to accelerated eGFR decline, while Toyama^[14]^ highlighted the renoprotective effects of SGLT2 inhibitors in slowing DKD progression. More recently, Xiao et al^[15]^ demonstrated that GV remained significantly associated with renal outcomes even in patients treated with SGLT2 inhibitors, though the adverse effect was attenuated (findings consistent with our subgroup results).

Mechanistically, GV may accelerate renal injury through several interrelated pathways. Recurrent glucose fluctuations trigger oxidative stress and inflammatory responses, promoting glomerulosclerosis and interstitial fibrosis.^[16]^ Gómez-Banoy et al^[17]^ demonstrated that higher GV is significantly associated with elevated inflammatory markers in patients with T2D and CKD. GV also contributes to glomerular basement membrane injury via enhanced formation of AGEs^[18]^ and induces vascular endothelial dysfunction, thereby aggravating proteinuria.^[19]^ Experimental studies suggest that intermittent glucose swings, compared with sustained hyperglycemia, more strongly promote endothelial apoptosis and oxidative damage.^[20]^ Animal models further indicate that GV accelerates tubular epithelial injury through mitochondrial dysfunction,^[6]^ while clinical evidence links GV to increased urinary inflammatory biomarkers.^[11]^ Moreover, GV may facilitate DKD progression by activating the renin–angiotensin–aldosterone system and altering renal hemodynamics.^[21]^ Collectively, these mechanisms provide a biological rationale for the associations observed in this study.

Although informative, this study has certain limitations. First, as a single-center retrospective design, it cannot establish causality and may be subject to selection bias. Second, GV indices were derived from CGM performed at varying time points, which may have introduced variability in data completeness and monitoring duration. Third, although extensive adjustments for confounders were made, residual influences from factors such as diet, physical activity, and medication adherence cannot be fully ruled out. Finally, the lack of renal histopathology or molecular data restricted deeper investigation into underlying mechanisms. Future prospective, multicenter studies with larger cohorts, complemented by imaging and molecular biomarkers, are warranted to validate the independent prognostic value of GV and to inform potential intervention strategies.

In summary, this study demonstrates that elevated GV is strongly associated with adverse renal outcomes in patients with DKD, with CV emerging as the most reliable predictor. The effect of GV was more pronounced in high-risk subgroups, underscoring its potential value in clinical risk stratification and individualized glycemic management. Integrating GV monitoring into comprehensive DKD care may help slow renal function decline and improve patient outcomes.

## Author contributions

**Conceptualization:** Ling Yang, Bo Zhou, Rensheng Deng.

**Data curation:** Ling Yang, Bo Zhou, Rensheng Deng.

**Formal analysis:** Ling Yang, Bo Zhou, Rensheng Deng.

**Funding acquisition:** Ling Yang, Bo Zhou, Rensheng Deng.

**Investigation:** Bo Zhou, Rensheng Deng.

**Writing – original draft:** Ling Yang, Bo Zhou, Rensheng Deng.

**Writing – review & editing:** Ling Yang, Bo Zhou, Rensheng Deng.
